# Mining tailings dam failures and human health: a scoping review of environmental exposure, potentially toxic elements, and epidemiological evidence, 2015–2025

**DOI:** 10.1007/s10653-026-03542-9

**Published:** 2026-10-22

**Authors:** Tamires Patricia Souza, Amanda Duim Ferreira, Douglas Gomes Viana, Juliet Emilia Santos de Sousa, Tiago Osório Ferreira

**Affiliations:** 1 Universidade de São Paulo, São Paulo, Brazil; 2 Universidade Estadual de Campinas, São Paulo, Brazil

**Keywords:** Potentially toxic elements, Environmental contamination, Epidemiological surveillance, Risk assessment, Environmental health

## Abstract

Mining is essential for economic development; however, the increasing number of tailings dam failures has become a major environmental and public health concern. This review aimed to characterize mining tailings dam disasters reported worldwide between 2015 and 2025 and summarize the available evidence on their human health impacts. A scoping review was conducted using Web of Science, Scopus, MEDLINE, Google Scholar, and institutional sources institutional reports from World Information Service on Energy (WISE), United Nations Environment Programme (UNEP), GRID-Arendal, and World Health Organization (WHO). Each identified disaster was individually investigated to compile information on disaster characteristics, released tailings, mineral commodities, potentially toxic elements (PTEs), and available epidemiological evidence. A total of 670 deaths and 112 injuries were reported, while approximately 75 people were reported missing. At least 728 displaced people and 55 displaced families were also reported. Gold, copper, and jade were the mineral commodities most frequently involved, while arsenic, lead, mercury, manganese, and zinc were the most frequently reported PTEs in the tailings associated with these disasters. Despite the large number of disasters, only two incidents (Mariana and Brumadinho) have been investigated in population-based studies evaluating human health following the events, revealing a major global knowledge gap.

**Supplementary Information:**

The online version contains supplementary material available at https://doi.org/10.1007/s10653-026-03542-9.

## Highlights

- 49 mining tailings dam disasters were identified worldwide between 2015 and 2025.
- Only two of 49 reported disasters have been investigated in population-based studies evaluating human health following the events.
- Gold was the mineral commodity most frequently involved in reported disasters.
- As and Pb were the most frequently reported PTEs, followed by Hg, Mn, and Zn.
- Tailings dam failures are associated with persistent environmental contamination and multiple health risks.
- Long-term health assistance should become a priority in regions affected by mining disasters.

**Supplementary Information:**

The online version contains supplementary material available at https://doi.org/10.1007/s10653-026-03542-9.

## Introduction

Mining plays a crucial role in global economic and technological development by providing essential raw materials for different sectors such as construction, energy, electronics, and manufacturing. The extraction of minerals and metals is fundamental to infrastructure development, technological advancements, and the transition to renewable energy systems (Jain et al., 2016), while also contributing to economic growth, job creation, and industrial innovation (Carvalho, 2017; David et al., 2016).

The contribution of the mining sector to national gross domestic product (GDP) varies substantially across countries, reflecting differences in the distribution of natural resources, economic structures, and levels of economic diversification. However, the economic benefits of the mining industry are accompanied by substantial environmental and safety concerns, particularly regarding the management of mining tailings dams. These operations may generate significant amounts of waste after ore extraction, with residue quantities varying by several orders of magnitude depending on the type of commodity (Nassar et al., 2022). In practical terms, this results in massive waste generation on a national and global scale. For example, in Brazil, projections by the National Mining Agency indicate that billions of tones of mining tailings could be produced by 2030, highlighting the scale of material requiring long-term management (Aredes et al., 2025; Luz et al., 2012). These wastes typically consist of finely crushed rock, sand, and process water used during mineral processing (Islam et al., 2021; Roche et al., 2017), and may contain potentially toxic elements capable of contaminating surrounding ecosystems, particularly when stored in tailing dams (Páez-Osuna et al., 2023).

The growing generation of mining waste, driven in part by declining ore grades, has led to the global expansion of tailings storage facilities (Rötzer & Schmidt, 2018). It is estimated that there are approximately 18,000 such facilities worldwide, including about 3,500 active structures, although these figures may be underestimated (Earthworks, 2023; Lyu et al., 2019). Given their scale, longevity, and structural complexity, concerns regarding their stability and the risk of failure have intensified.

The collapse of mining dams represents one of the most serious consequences of these systems, causing widespread environmental degradation and exposing humans, animals, and ecosystems to potentially toxic elements (PTEs) (Hudson-Edwards et al., 2024; Kossoff et al., 2014). Recent failures have resulted in the contamination of water bodies, loss of biodiversity, and long-term soil degradation, while also disrupting livelihoods and access to essential resources in affected communities (Beck et al., 2020; Stewart, 2020). The Mariana (2015) and Brumadinho (2019) disasters in Brazil exemplify these impacts, including prolonged consequences for public health; the latter alone caused 272 deaths, representing the country’s deadliest workplace accident (Freitas et al., 2022; Peixoto et al., 2022).

These risks are further exacerbated by climate change, as increasing climate variability and extreme weather events can compromise dam stability and increase the mobilization of PTEs and other constituents from tailings (Bolan et al., 2024; Hudson-Edwards et al., 2024; Roche et al., 2017). Consequently, exposure to potentially toxic elements, such as aluminum (Al), arsenic (As), cadmium (Cd), lead (Pb), and manganese (Mn), has been reported in affected populations, with documented associations ranging from dermatological conditions to cardiovascular and neurodegenerative consequences (Boim et al., 2020; Cavalheiro Paulelli et al., 2022; Mitra et al., 2022; Vormittag et al., 2018).

Despite the growing evidence, significant gaps remain in the understanding of the long-term and integrated impacts of mining disasters on human health. Current studies are often fragmented, with limited integration between environmental contamination data and epidemiological outcomes, particularly in regions with expanding mining activity and diverse regulatory and governance contexts.

This study aims to address these gaps by systematically mapping tailings dam disasters occurring between 2015 and 2025 and characterizing the available evidence on their human health implications. Evidence from recent decades indicates not only an increase in the number of failures worldwide, but also a broader geographic distribution of accidents across multiple countries. The 2010s presented the highest average casualty risks reported since the 1960s, with Brazil and Myanmar showing substantial reported losses associated with tailings dams failures (Fell et al., 2000; Halabi et al., 2022). Current trends also suggest that this pattern may continue into the 2020s (WISE, 2025). By focusing on long-term exposure to potentially toxic elements, this review integrates environmental and epidemiological evidence to better characterize risk pathways and inform prevention and mitigation strategies on a global scale.

## Methods

This study was conducted as a scoping review to map mining tailing dam disasters reported worldwide and characterize the available evidence on their impacts on human health. The scoping review approach was selected because the available evidence is heterogeneous and includes peer-reviewed studies, technical reports, institutional documents, and other gray literature. The review was conducted and reported in accordance with the Preferred Reporting Items for Systematic Reviews and Meta-Analyses extension for Scoping Reviews (PRISMA-ScR).

The review addressed the following questions: (i) What mining tailings dam disasters were reported worldwide between 2015 and 2025? And (ii) What evidence is available regarding their impacts on human health? The primary objective was to map mining disasters reported worldwide between 2015 and 2025. The secondary objective was to characterize the available evidence on human health impacts associated with these events, including documented human exposure, health outcomes, and the timing and characteristics of post-disaster health assessment.

Information on potentially toxic elements (PTEs) reported in tailings or associated environmental media was extracted when available to contextualize potential exposure pathways relevant to human health.

### Search strategy

**Fig. 1:**
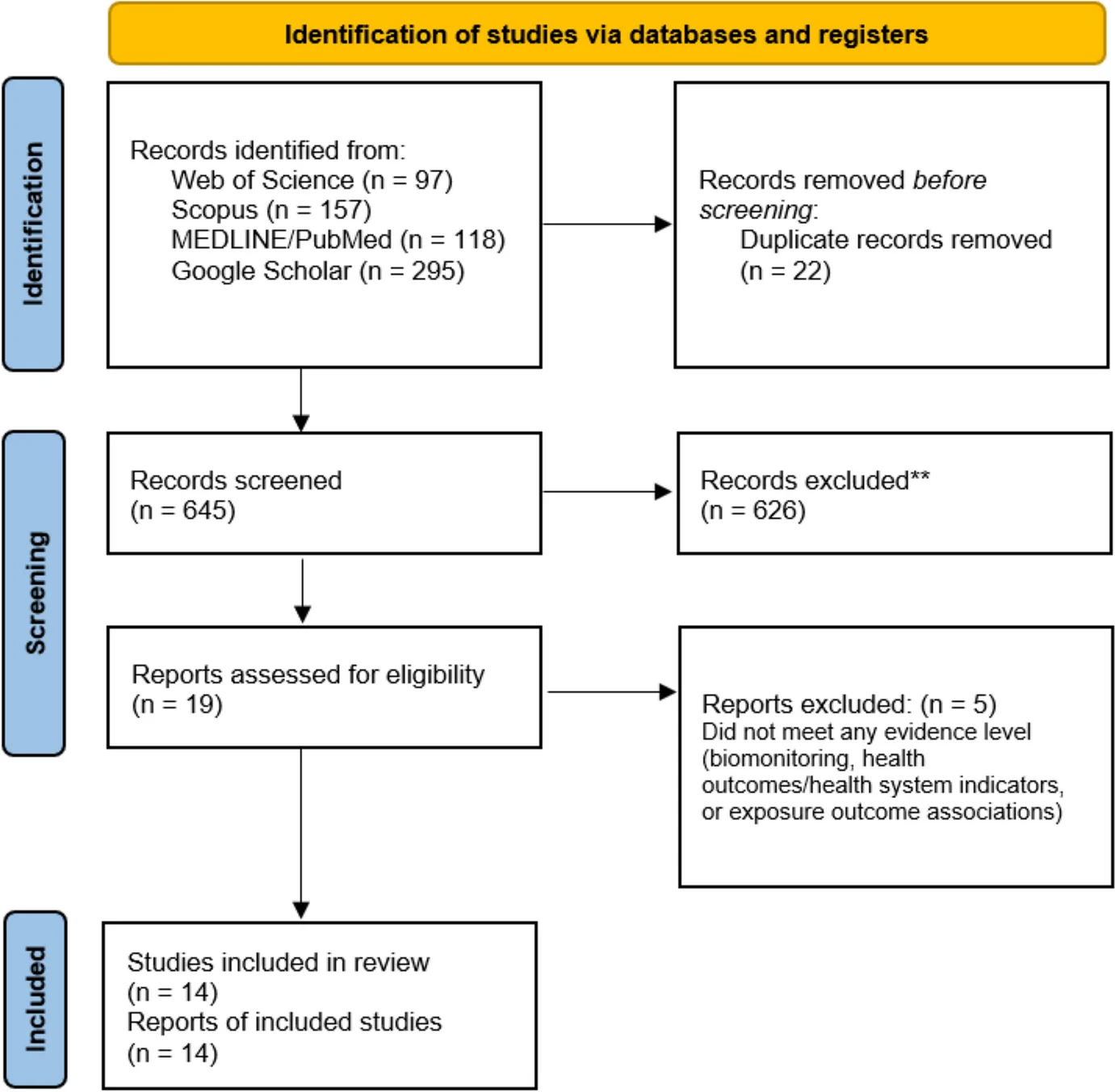
PRISMA-ScR flow diagram showing the identification, screening, eligibility assessment, and inclusion of studies evaluating human health following mining tailings dam disasters. Studies were identified through Web of Science, Scopus, MEDLINE/PubMed, and Google Scholar, complemented by targeted event searches and supplementary sources. Following screening and eligibility assessment, 14 studies were included in the final evidence synthesis, covering two tailings dam disasters, Mariana and Brumadinho, among the 49 disasters identified between 2015 and 2025

A two-step search strategy was used. First, a structured database search was conducted to identify studies addressing mining dam disasters and human health. Searches were performed in Web of Science, Scopus and MEDLINE, complemented by Google Scholar, using a combination of terms related to tailings dams, tailings storage facilities, dam failure, mining disasters, human exposure, and human health. The search was not restricted by language or publication year. The 2015–2025 temporal range was subsequently applied during the identification and selection of eligible tailings dam disasters for the present review (Fig. 1). The complete database search strategies, including Boolean operators, truncation and wildcard characters, field restrictions, applied limits, and search dates, are provided in Supplementary Table S1.

Due to the structured database search identified a limited number of studies directly addressing human health impacts following individual tailing dam disasters, a complementary search was subsequently performed. Each disaster identified in the event inventory was searched individually using the disaster name, location, mining commodity, and health related terms.

Additional evidence was identified through Google Scholar, institutional websites, gray literature sources, and list of references. This iterative approach was used to identify health-related studies that might not have been retrieved through generic database searches because they were indexed under the name of the disaster, affected municipality, mining project, river basin, or other event-specific terminology rather than under generic terms such as “tailings dam failure.”

For the identification and verification of disaster events, the World Information Service on Energy (WISE) (2025) Uranium Project chronology of major tailings dam failures was used as the primary source, complemented by documents from the United Nations Environment Programme (UNEP) (2025), GRID-Arendal (2024), the World Health Organization (WHO) (2024), scientific databases, and other relevant institutional sources. Each candidate event was subsequently investigated individually and cross-checked against available sources. Multiple reports referring to the same disaster were consolidated into a single event record.

Gray literature was searched because information on mining disasters and post-disaster health assessments is frequently reported outside the peer-reviewed literature. Relevant documents were identified through WISE (2025), UNEP (2025), GRID-Arendal (2024), WHO (2024), government and institutional websites, and targeted web searches using combinations of disaster-specific terms and health-related keywords. Google Scholar was used as a supplementary source to identify additional peer-reviewed and gray-literature documents not retrieved in the primary bibliographic databases. All results retrieved for each targeted search were screened for relevance according to the predefined eligibility criteria.

### Inclusion and exclusion criteria

Disaster events were eligible for inclusion criteria when they involved a mining tailings dam or tailings storage facility and were identified as a documented tailings dam disaster. The event inventory was not restricted to a specific failure mechanism. Therefore, different types of documented tailings dam disasters were considered during the identification of events, and the mechanism or type of event was recorded as reported in the available sources.

Studies were eligible for inclusion if they evaluated human health directly affected by a mining tailing dam disaster. Studies addressing populations affected by the disaster were considered eligible when they assessed human exposure, health outcomes, or associations between exposure and health outcomes following the event.

Studies were excluded if they did not evaluate human health directly affected by a tailings dams disaster, including studies focused on environmental contamination, geochemical characterization, ecological impacts, or toxicological effects unrelated to populations directly affected by the disaster. Studies addressing potential health effects without evaluating populations directly affected by a specific tailing dam disaster were considered contextual evidence and were not included as direct post-disaster health evidence.

### Data extracted

For each disaster, the following information was extracted: date, location, country, mineral commodity, incident type, estimated volume of tailings released, reported deaths, injuries, missing persons, and displacement, and available information on human health impacts. Where available, information on PTEs environmental contamination, human exposure, and exposure pathways was also recorded to contextualize the reported health evidence.

For sources related to health, the following variables were extracted: study design, population characteristics, occupational or residential exposure status, sample size, exposure assessment, biological and environmental measurements, comparison group, time since the disaster, health outcomes, principal findings, confounders and statistical adjustment, and reported limitations. Occupational and environmental exposure were recorded separately whenever this information was available.

### Evidence classification

To facilitate the interpretation of heterogeneous health related evidence, findings from studies involving populations affected by tailings dam disasters were classified according to the type of evidence provided. Based on the evidence identified in the eligible studies, three levels were used: Level 1, biomonitoring evidence; Level 2, documented health outcomes and health system indicators; and Level 3, exposure outcome associations.

Level 1 included studies assessing internal exposure or biological responses through human biomonitoring, such as measurements of potentially toxic elements or biomarkers in blood or urine. Level 2 included studies reporting directly assessed health outcomes or health system indicators, such as symptoms, diagnosed diseases, mental health conditions, multimorbidity, respiratory outcomes, hospitalization, mortality, psychotropic drug use, or healthcare utilization. Level 3 included studies in which statistical analyses examined associations between exposure or exposure related variables and health outcomes, biomarkers, or health system indicators.

A single study could be classified into more than one level when it provided different types of evidence (Fig. 1). The classification was intended to characterize the type and directness of the available evidence and was not used as a measure of study quality, risk of bias, or causal certainty. No included study provided sufficient evidence to support causal inference.

## Results and discussion

### Characteristics of the major tailings dam failures

Over the past decade (2015–2025), 49 mining tailings dam disasters have been officially recorded worldwide, of which, 6 were due to overflow and 43 to tailing dam failures (Table 1). Reported tailings volumes varied substantially across the disaster inventory, and volume estimates were unavailable or reported using different units for several events. Accordingly, no standardized aggregate volume was calculated. Volumes of each specific event are provided in Table 1. The available estimates illustrate the large and highly variable magnitude of tailings releases associated with these disasters. Considering that tailings may contain potentially toxic elements, fine particulate matter, and processing chemicals (Kossoff et al., 2014), the magnitude of these releases reinforces the need for long-term environmental and health monitoring programs.

**Table 1:** Characteristics of the past decade of mining tailing disasters

Date	Location	Ore type	Type of accident	Reported tailings volume	Potential Toxic Elements	Studies on human health directly affected	Direct human impacts
2025, Mar. 22	Bahodopi District, Central Sulawesi, Indonesia	Nickel	Failure	Not estimated	Ni, Co, Mn, Cu, Cr, Pb, As	Not found in literature	Three workers killed
2025, Mar. 16	Potosí, Bolivia	Tin	Failure	Not estimated	As, Pb, Zn, Fe, Mn	Not found in literature	Two killed
2025, Feb. 18	Kalulushi, Zambia	Copper	Failure	Not estimated	As, Pb, Cd, Hg, Zn, Cr, Mn	Not found in literature	Not reported
2025, Feb. 17	Western Region, Ghana	Gold	Failure	80,000 m^3^	As, Pb, Hg, CN, Ni, Zn, Cu, Cd, Cr	Not found in literature	Not reported
2025, Jan. 13	Kachin state, Myanmar	Jade	Failure	Not estimated	Not reported	Not found in literature	12 killed ≃ 50 missing
2024, Dec. 7	Conceição do Pará, Minas Gerais, Brazil	Gold	Failure	750,000 m^3^	As, Pb, Hg, CN, Ni, Zn, Cu, Cd, Cr	Not found in literature	255 displaced
2024, June 13	Valparaíso region, Chile	Copper	Failure	Not estimated	As, Pb, Cd, Hg, Zn, Cr, Mn	Not found in literature	Not reported
2024, May 12	Kachin state, Myanmar	Jade	Failure	Not estimated	Not reported	Not found in literature	≃ 25 missing
2024, May 11	Surigao Del Norte Province, Philippines	Gold	Failure	Not estimated	Not reported	Not found in literature	55 families displaced
2024, Jan. 25	Kachin, Myanmar	Jade	Failure	3 million m^3^	Not reported	Not found in literature	Not reported
2023, Nov. 4	Imbabura, Ecuador	Gold	Failure	Not estimated	As, Pb, Hg, CN	Not found in literature	Not reported
2023, Jan. 31	Fort Chipewyan, Alberta, Canada	Bitumen	Overflow	5,300 m^3^	Not reported	Not found in literature	Not reported
2022, Nov. 7	Kishapu District, Shinyanga Province, Tanzania	Diamond	Failure	12.8 million m^3^	Not reported	Not found in literature	Three injured 115 displaced
2022, Sep. 11	Xhariep, Free State, South Africa	Diamond	Failure	5.04 million m^3^	Not reported	Not found in literature	Two killed 76 injured ≃ 300 displaced
2022, Jul. 23	Agua Dulce, Potosí, Bolivia	Silver Zinc	Failure	5 million m^3^	Not reported	Not found in literature	Not reported
2022, May	Fort Chipewyan, Alberta, Canada	Bitumen	Overflow	53,000 m^3^	Not reported	Not found in literature	Not reported
2022, Mar. 27	Jiaokou County, Shanxi Province, China (SU, 2023)	Bauxite	Failure	224,000 m^3^	Not reported	Not found in literature	Not reported
2022, Jan. 20	Sambalpur district, Odisha (formerly Orissa), India	Iron	Failure	Not estimated	Not reported	Not found in literature	One displaced
2022, Jan. 8	Nova Lima, Minas Gerais, Brazil	Iron	Overflow	85,000 m^3^	Not reported	Not found in literature	Five killed
2021, Dec. 24	Ulundi, KwaZulu-Natal, South Africa	Coal	Failure	1,500 m^3^	Not reported	Not found in literature	Not reported
2021, Nov. 26	San Antonio de Putina province, Puno, Peru	Gold	Failure	Not estimated	Not reported	Not found in literature	Not reported
2021, Nov. 18	Sebinkarahisar district of Giresun, Turkey	Lead Zinc Copper	Failure	> 4,500 t	Not reported	Not found in literature	Not reported
2021, July 27	Saurimo, Lunda Sul, Angola	Diamond	Failure	35 million m^3^	Not reported	Not found in literature	Not reported
2020, July 2	Hpakant, Kachin state, Myanmar	Jade	Failure	30 m^3^	Not reported	Not found in literature	126 killed 31 injured
2020, May 1	Canelas, Durango, Mexico	Lead Zinc	Failure	6,000 m^3^	Not reported	Not found in literature	Not reported
2020, Mar. 28	Yichun City, Heilongjiang Province, China	Molybdenum	Overflow	2.53 million m^3^	Not reported	Not found in literature	Not reported
2019, Nov. 13	San Antonio de Putina province, Puno, Peru	Gold	Failure	Not estimated	Not reported	Not found in literature	Not reported
2019, Oct. 1	Nossa Senhora do Livramento, Mato Grosso, Brazil	Gold	Failure	582,171 m^3^	Not reported	Not found in literature	Not reported
2019, July 10	Churcampa province, Huancavelica region, Peru	Copper	Failure	67,488 m^3^	Not reported	Not found in literature	Not reported
2019, Apr. 22	Hpakant, Kachin state, Myanmar	Jade	Failure	Not estimated	Not reported	Not found in literature	59 killed
2019, Apr. 9	Muri, Jharkhand, India	Bauxite	Failure	Not estimated	Not reported	Not found in literature	Not reported
2019, Mar. 29	Oriente Novo, Rondônia, Brazil	Tin	Failure	Not estimated	Not reported	Not found in literature	Not reported
2019, Jan. 25	Brumadinho, Região Metropolitana de Belo Horizonte, Minas Gerais, Brazil	Iron	Failure	12 million m^3^	Fe, Al, Mn, Zn, Cu, Pb, Cd and U	Peixoto et al., 2022 Maluf et al., 2022 Nascimento-Souza et al., 2022 Garcia et al., 2022 Loyola et al., 2022 Campos et al., 2022 Mota et al., (2021 Macinko et al., 2022 Santos et al., 2023 Vidal et al., 2024	267 killed
2018, June 4	Urique, Chihuahua, Mexico	Gold Silver	Failure	249,000 m^3^	Not reported	Not found in literature	Three killed Two injured Four displaced
2018, Mar. 9	Cadia, New South Wales, Australia	Gold Copper	Failure	1.33 million m^3^	Not reported	Not found in literature	Not reported
2018, Mar. 3	Recuay province, Áncash region, Peru	Not found in literature	Failure	80,000 m^3^	Not reported	Not found in literature	Not reported
2018, Feb. 17	Barcarena, Pará, Brazil	Bauxite	Overflow	Not estimated	Not reported	Not found in literature	Not reported
2017, Sep. 17	Bong County, Liberia	Gold	Failure	11,500 m^3^	Not reported	Not found in literature	Not reported
2017, June 30	Mishor Rotem, Israel	Phosphate	Failure	100,000 m^3^	Not reported	Not found in literature	Not reported
2017, Mar. 12	Hubei province, China	Copper Gold Silver Iron	Failure	200,000 m^3^	Not reported	Not found in literature	Two killed One displaced
2016, Dec. 28	Hpakant, Kachin state, Myanmar	Jade	Failure	Not estimated	Not reported	Not found in literature	50 displaced
2016, Oct. 27	Itogon, Benguet province, Philippines	Gold	Overflow	50,000 t	Not reported	Not found in literature	Not reported
2016, Aug. 27	Mulberry, Polk County, Florida, USA	Phosphate	Failure	840,000 m^3^	Not reported	Not found in literature	Not reported
2016, Aug. 8	Luoyang, Henan province, China	Bauxite	Failure	2 million m^3^	Not reported	Not found in literature	Farmers and domestic animals killed (no number specified)
2016, Aug. 4	Tamarugal Province, Tarapacá Region, Chile	Copper Molybdenum	Failure	4,500 m^3^	Not reported	Not found in literature	Not reported
2016, May 22	Ridder, Kazakhstan	Zinc	Failure	Not estimated		Not found in literature	Not reported
2015, Nov. 23	Longnan city, Gansu province, China	Antimony	Failure	25,000 m^3^	As, Hg and Sb	Not found in literature	Not reported
2015, Nov. 21	Hpakant, Kachin state, Myanmar	Jade	Failure	Not estimated	Not reported	Not found in literature	172 killed
2015, Nov. 5	Bento Rodrigues, distrito de Mariana, Minas Gerais, Brazil	Iron	Failure	50 million m^3^	Ba, Pb, As, Sr, Fe, Mn and Al	Vormittag et al., 2018 Motta et al., 2021 Paulelli et al., 2022 Paulelli et al., 2023 Vidal et al., 2024	17 killed Two displaced

Tailings dam failures were reported across multiple regions worldwide, with the majority located in Asia (41%), followed by South America (33%), Africa (14%), and North America (10%), and were associated with significant human consequences, including deaths, injuries, and population displacement. According to the available records, 670 deaths and 112 injuries were reported, along with at least 728 displaced people and 55 displaced families. The geographical distribution of reported disasters may partly reflect the intensity of mining activities and the number of tailings dams in different regions. Previous studies have identified factors such as inadequate maintenance, infrastructure conditions, operational practices, and regulatory and governance challenges as potential contributors to tailings dam failures (Halabi et al., 2022; Stark & Eid, 2022); however, these factors were not systematically assessed in the present study.

**Fig. 2:**
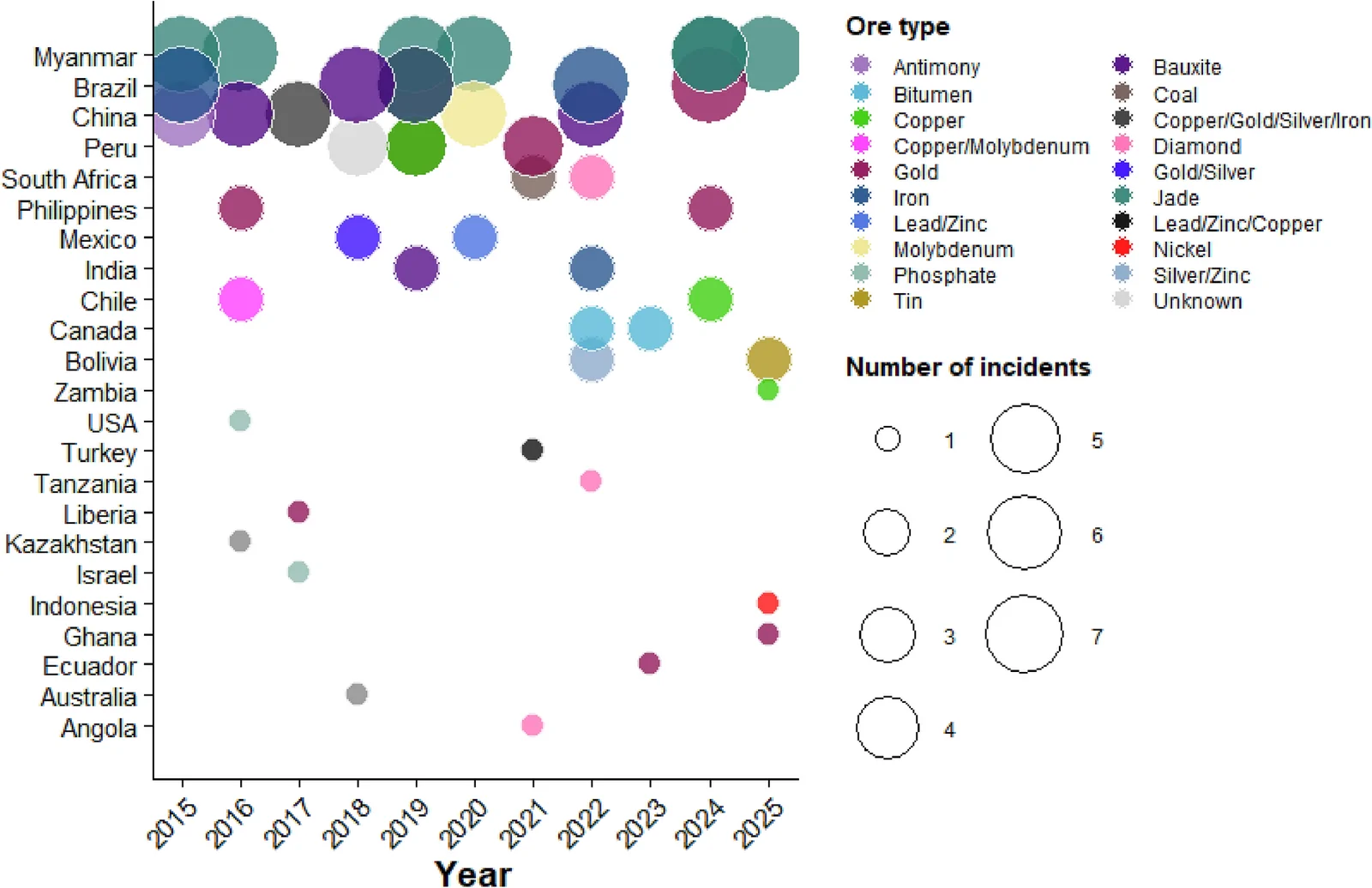
Spatial and temporal distribution of reported tailings dam disasters, 2015–2025. Bubble size represents the number of incidents reported for each country/year combination, and colors indicate the associated mineral commodity

Modified from WISE Uranium Project, 2025. Available at: https://www.wise-uranium.org/mdaf.html. As some records of the events were incomplete or inconsistent for certain disasters, the aggregate figures relating to displacement, missing persons and the volume of waste should be interpreted as minimum or approximate estimates, based on the available sources. The spatial distribution of disasters revealed geographical clusters of reported tailings dam disasters, particularly in regions with intensive extractive activities, including Minas Gerais (Brazil), Kachin State (Myanmar), Potosí (Bolivia), mining regions in China, and gold- and copper-producing areas in Peru and Mexico (Fig. 2). These regions contain substantial mining activity and multiple tailings storage facilities, which may partly explain the concentration of reported events in the review inventory (Halabi et al., 2022; Lyu et al., 2019).

The observed geographic distribution may reflect differences in mining activity, the number of tailings storage facilities, and regional characteristics of tailings dam management; however, these factors were not systematically evaluated in the present study. Previous studies have identified inadequate maintenance, aging infrastructure, operational deficiencies, and regulatory and governance challenges as potential contributors to tailings dam failures worldwide (Azam & Li, 2010; Stark & Eid, 2022).

Factors such as regulatory and governance challenges, inadequate oversight and monitoring, aging infrastructure, insufficient maintenance, and operational deficiencies have been identified as potential contributors to tailings dam failures (Azam & Li, 2010; Lyu et al., 2019; Stark & Eid, 2022). Socioeconomic conditions, including limited resources for disaster preparedness and recovery, may further influence the consequences experienced by affected communities (Azam & Li, 2010; Lyu et al., 2019; Stark & Eid, 2022). However, these factors were not systematically evaluated in the present study and therefore cannot be considered explanatory variables for the spatial patterns observed in our dataset.

**Fig. 3:**
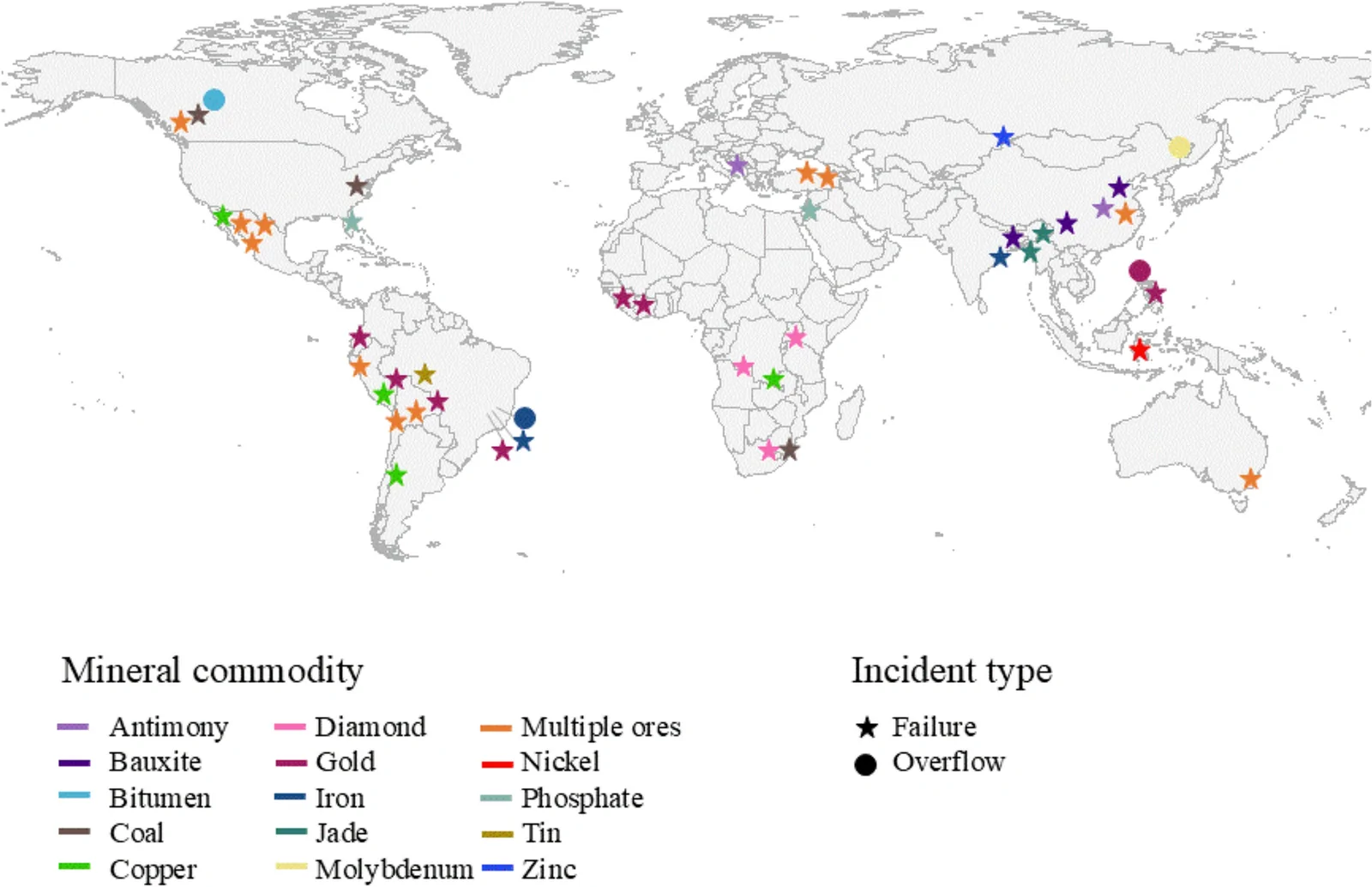
Spatial distribution of reported tailings dam disasters worldwide, 2015–2025, classified by incident type and mineral commodity. Symbols indicate incident type (Failure or Overflow), and colors indicate the associated mineral commodity

Of the 49 tailings dam disasters identified in this review between 2015 and 2025, 43 (87.8%) were classified as failures and 6 (12.2%) as overflows (Fig. 3). These figures describe the distribution of incident types within the disaster inventory compiled for this review and should not be interpreted as the proportion of all disasters related to mining worldwide. Gold was the most frequently reported mineral commodity, occurring in 12 of the 49 disasters (24.5%), followed by copper and jade, each reported in 7 events (14.3%), and iron in 5 events (10.2%). These commodities were associated with the reported occurrence of multiple PTEs in the tailings (Charles et al., 2013; Clark et al., 2021; Mensah et al., 2020a, 2020b).

**Fig. 4:**
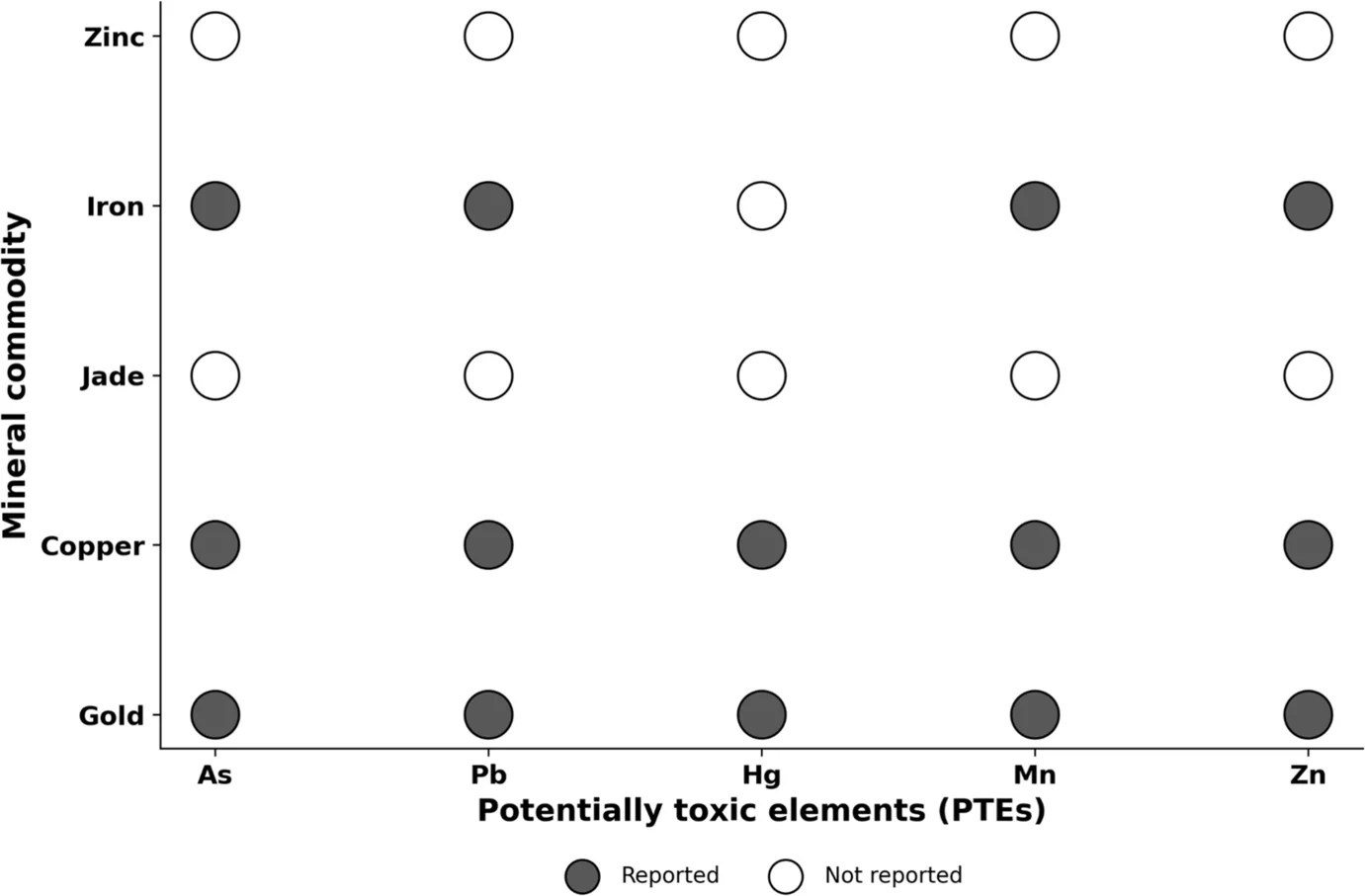
Occurrence of the five most frequently reported potentially toxic elements (PTEs) across the five major mineral commodities involved in tailings dam disasters between 2015 and 2025. Filled circles indicate that the respective PTE was reported in the mining tailings of at least one disaster involving that mineral commodity, whereas empty circles indicate that no occurrence was identified among the disasters included in this review. The figure illustrates occurrence patterns only and should not be interpreted as evidence of exclusive or direct associations between individual commodities and specific PTEs

Potential Toxic Elements information was reported for 10 of the 49 disasters included in the review. Among these events, arsenic (As) and lead (Pb) were the most frequently reported PTEs, each identified in 9 of the 10 events, followed by mercury (Hg), manganese (Mn), and zinc (Zn), each reported in 6 events (Table 1). These frequencies describe documented reporting among events with available PTE information. Events for which PTE information was not reported were not interpreted as lacking these elements. As shown in Fig. 4, these major mineral commodities were associated with multiple PTEs rather than a single element, highlighting the chemical complexity of mining tailings and the potential for simultaneous exposure to mixtures of hazardous elements.

The occurrence of multiple PTEs across mining tailings of different mineral commodities reflects the complex geochemical composition of mining ores and processing residues. Although the presence of the PTE in the mining tailings does not directly translate into environmental contamination, mining is frequently associated with both occurrence and release of PTEs that can become environmental contaminants, such as As and Hg in gold mining. Geological mineral associations (Mensah et al., 2020a, 2020b; Torrance et al., 2021), the extraction and processing pathways, and secondary environmental mobilization mechanisms can be responsible for turning PTEs contained in mine ores and mine tailings into environmental contaminants.

For instance, arsenic can be a constituent of some gold ores, particularly in low-sulphide gold-quartz vein deposits (Mensah et al., 2020a, 2020b). High background concentrations of arsenic can occur in gold deposits worldwide, with arsenic concentrations in refractory ores ranging from trace levels to over 5,000 mg kg^−1^ (Seitkan & Redfren, 2021; Torrance et al., 2021). Also, excavation, physical crushing, chemical extraction, and thermal treatments can accelerate the release of arsenic from solid rock (Thiombane et al., 2023) to surrounding soils, water systems, and the atmosphere, where they can become contaminants (Da Silva Faria et al., 2023; Kastury et al., 2024; Tabelin et al., 2020). For instance, in artisanal mining, Hg may also be intentionally used during gold extraction through amalgamation (Charles et al., 2013; Clark et al., 2021).

Once released from primary ores, PTEs migrate through air, water, and soil via distinct geochemical processes (Da Silva Chen et al., 2023; Faria et al., 2023). For instance, weathering alters primary arsenopyrite into secondary iron oxyhydroxides, which retain arsenic via adsorption or co-precipitation (Chen et al., 2023; Zha et al., 2025). However, this retention is highly sensitive to environmental conditions; fluctuating hydrological regimes and flooding trigger the reductive dissolution of iron oxyhydroxides, desorbing bound arsenate and reducing it to more mobile and toxic arsenite (Marrugo-Negrete et al., 2024). Alkaline pH conditions further accelerate arsenic desorption into groundwater and riverine networks (Seitkan & Redfren, 2021; Tabelin et al., 2020). Consequently, mine-derived arsenic bioaccumulates across terrestrial and aquatic ecosystems, contaminating drinking water and which can lead to severe human health hazards (Da Silva Eisler et al., 2004; Faria et al., 2023).

In other cases, the mining tailings are composed of elements with low toxicity (e.g., iron present in iron oxides), but with high capacity to adsorb other PTEs (Queiroz et al., 2022), potentially poisoning risks to environmental and human health. Mine tailings composed of secondary iron (oxyhydr)oxides such as ferrihydrite, goethite, and schwertmannite act as primary scavengers that immobilize PTEs through surface complexation, surface precipitation, and structural coprecipitation (Baleeiro et al., 2018; Mertz et al., 2001; Ribeta et al., 1995). However, when mine tailings-impacted soils or sediments encounter suboxic or anoxic environments, dissimilatory iron-reducing microorganisms couple organic carbon oxidation to the reduction of solid Fe(III) phases to soluble Fe(II) (Keim et al., 2021; Ribeta et al., 1995; Zhang et al., 2025). The reduction of the underlying mineral substrate destroys reactive sorption sites, driving the rapid release of both Fe(II) and previously adsorbed or incorporated metals into pore solution (Davranche et al., 2000; Palau et al., 2021; Ribeta et al., 1995). Experimental studies demonstrate that when iron-rich tailings enter reducing settings, the release of metals occurs rapidly and scales with mineral crystallinity and organic matter availability (Barcellos et al., 2022). For instance, in laboratory anoxic incubations of soils affected by Fe-rich mine tailings, anoxic conditions triggered rapid iron reduction within 20 days alongside substantial increases in extractable concentrations of Cd, Co, Cr, Cu, Mn, Ni, Pb, and Zn (Barcellos et al., 2022). It is also important to note that micronutrients such as Mn and Cu were among the five most frequently reported PTEs in tailings associated with the mining dam disasters. Although Mn and Cu are essential micronutrients, elevated exposure to these elements can be harmful to living organisms (Queiroz et al., 2022).

In response to this growing concern, several viable alternatives have been proposed to reduce or replace the reliance on traditional tailings dams in mining operations. The adoption of dry stacking tailing, for example, significantly decreases the risk of dam failure by removing excess water from tailings before stacking them in compacted layers, thereby enhancing stability and minimizing environmental impacts (Edraki et al., 2014; Kossoff et al., 2014). Dry stacking tailing is extremely relevant for commodities such as iron and aluminum, due to the high volume of ore processed and tailings produced.

Another possible solution is the implementation of paste tailings, which involves thickening the tailings to form a non-segregating slurry with minimal water content, thus improving the stability of the storage facility and decreasing water usage (Barreda et al., 2021; Li et al., 2022; Tariq & Yanful, 2013).

Additionally, the reuse and repurposing of tailings in the construction of building materials, such as bricks or concrete, offer a sustainable approach to waste management by reducing the volume of tailings requiring long-term storage (Xie et al., 2020). Advanced technologies, such as real-time monitoring systems and the application of artificial intelligence in risk assessment have been proven to play an important role in enhancing the safety and management of tailings dams (Forte et al., 2021). Because exposure to some of these PTEs has been associated with adverse health outcomes, the epidemiological evidence available for populations affected by major tailings dam failures is discussed in the following section.

## Health impacts of tailings dam failures

### Evidence on human health following tailing dam disasters

Among the 49 disasters identified between 2015 and 2025, 15 disasters reported deaths, injuries, or displacement of individuals. However, studies evaluating health related outcomes or human exposure following a specific disaster were identified for only two events: the Fundão dam failure in Mariana, Brazil, in 2015, and Córrego do Feijão dam failure in Brumadinho, Brazil, in 2019 (Table 2). The available evidence was heterogeneous and included self reported health outcomes, mental health assessments, clinical, and laboratory indicators, human biomonitoring, and analyses of health services data. The evidence also varied from measurements of internal exposure to statistical associations between exposure proxies and health outcomes.

**Table 2:** Health related evidence identified among populations affected by the Mariana and Brumadinho tailings dam disasters

Disaster	Study and design	Population/ sample	Health and environmental exposure	Study timing	Health outcome/measure	Main findings	Evidence level(s)	Confounders / statistical adjustment	Key limitations
Brumadinho, Brazil, 2019	Peixoto et al., 2022—prospective population-based cohort	Residents aged ≥12 years; n=3,080	Brumadinho population; comparison with Metropolitan Region of Belo Horizonte and Minas Gerais	2021 (~ 2 years post-disaster)	Self-rated health and diagnosed diseases	Hypertension (30.1%), high cholesterol (23.1%), and depression (22.5%) were among the most frequently reported diagnosed conditions	2	Descriptive analysis; comparison with 2019 National Health Survey data	No individual pre-disaster baseline; external population comparisons may differ in timing and characteristics
	Maluf et al., 2022—cross-sectional	Residents aged ≥12 years; n=3,080, with laboratory results available for 2,782	Non-exposed, directly affected, and residents of mining areas	2021 (~ 2 years post-disaster)	Hematological, lipid, hepatic and renal parameters; hs-CRP	Most laboratory parameters did not differ significantly between groups. Residents of mining areas had higher prevalence of altered HDL-C, hs-CRP, and eGFR <60 mL/min/1.73 m^2^	2–3	Multivariable Poisson regression adjusted for age, sex, diabetes, BMI, smoking, and hypertension; survey design considered	Cross-sectional design; exposure mainly based on residential categories rather than individual contaminant measurements
	Nascimento-Souza et al., 2022—cross-sectional	Adults; n=2,777	Residential area according to direct impact of dam failure	2021 (~ 2 years post-disaster)	Multimorbidity	Multimorbidity prevalence was 53.8%. Directly affected residence was associated with multimorbidity (adjusted OR 1.6; 95% CI 1.3–2.0)	2–3	Multiple logistic regression adjusted for sex, age, education, skin color, and residence area	Cross-sectional design; multimorbidity and exposure based partly on self-report and residential categories
	Garcia et al., 2022—cross-sectional	Adults; n=2,740	Sociodemographic characteristics and place of residence, including mining area	2021 (~ 2 years post-disaster)	Depression, PTSD, anxiety, thoughts of death/self-harm, sleep quality	Depressive symptoms (29.3%), PTSD (22.9%), and anxiety symptoms (18.9%) were prevalent. Female sex and residence in mining areas were positively associated with several psychiatric outcomes	2–3	Adjusted logistic regression including sex, age, marital status, skin color, education, and residence	Cross-sectional design; no pre-disaster psychiatric assessment; possible influence of other concurrent factors
	Loyola et al., 2022—cross-sectional	Adults; n=2,805	Exposure characteristics, including residence in directly affected and mining areas and disaster related losses	2021 (~ 2 years post-disaster)	Use of antidepressants and anxiolytics/hypnotics/sedatives	Psychotropic drug use was reported by 17.2% of participants. Use was higher among participants reporting disaster-related losses or worse perceived health and, for anxiolytics/sedatives, among residents of directly affected or mining areas	2–3	Pearson’s chi-square with Rao–Scott correction; no multivariable adjustment	Cross-sectional design; psychotropic use self-reported for the previous 15 days
	Campos et al., 2022—cross-sectional	Adults; n=2,772	Area of residence, including directly affected and mining areas; other covariates	2021 (~ 2 years post-disaster)	Asthma, COPD, wheezing, dry cough, nasal irritation	Prevalence was 7.2% for asthma, 3.5% for COPD, 8.8% for wheezing, 23.6% for dry cough, and 31.8% for nasal irritation. Residence in directly affected or mining areas was associated with asthma; residents of affected areas reported greater odds of respiratory symptoms	2–3	Crude and adjusted logistic regression; models included sociodemographic, smoking, occupational, residence, and exposure variables	Cross-sectional design; exposure mainly represented by geographic/indirect measures
	Mota et al., 2022—cohort baseline analysis	Residents aged ≥12 years; n=3,080	Geographic strata and sociodemographic/ exposure-related characteristics	2021 (~ 2 years post-disaster)	Urinary As, Cd and Hg; blood Pb and Mn	Proportions above reference values were 38.1% for Mn, 33.4% for As, 5.0% for Pb, 0.8% for Hg, and 0.2% for Cd	1–3	Multiple logistic regression adjusted for age, sex, skin color, income, smoking, and residence; survey design considered	Cross-sectional biomonitoring; timing and sources of exposure cannot be established
	Macinko et al., 2022—cross-sectional analysis	Adults; n=2,805	Exposed vs. non-exposed population and mining-community comparison group	2021 (~ 2 years post-disaster)	Medical consultation, hospitalization, usual source of care, preventive care and vaccination	The exposed group had a higher prevalence of medical consultation. Several healthcare indicators did not differ significantly between exposure groups	2–3	Weighted robust Poisson regression with multivariable adjustment for sociodemographic, socioeconomic, insurance, and health factors	Cross-sectional design; geographic exposure categories and self-reported healthcare indicators may leave residual confounding
	Santos et al., 2023—cross-sectional	Children aged 0–6 years; n=217, urine samples from 197	Residence in directly and non-directly affected locations	August 2021 (~ 2.5 years post-disaster)	Urinary As, Cd, Pb, Hg, and Mn	Urinary As and Mn concentrations were higher in some non-directly affected locations than in directly affected areas. No clinical health outcome was assessed	1	Descriptive comparisons; no multivariable adjustment	Small pediatric sample; residential location used as an exposure proxy; no clinical outcome assessed
Mariana, Brazil, 2015	Vormittag et al., 2018—exploratory cross-sectional descriptive study	Residents of Barra Longa; n=507	Population affected by the disaster	Post-disaster assessment	Self-reported physical, mental and social health	Thirty-seven percent reported worse health than before the disaster. Reported health problems included respiratory conditions (40%), skin conditions (15.8%), and behavioral disorders (11%)	2	Descriptive analysis; no multivariable adjustment	Exploratory cross-sectional study; self-reported outcomes and no individual exposure measurement or pre-disaster clinical baseline
	Motta & Borges, 2021—mixed-methods occupational study	164 mining workers; 25 also participated in qualitative interviews	Workers in Mariana vs. miners in Conceição do Mato Dentro	Post-disaster assessment	Common mental disorders and psychosocial conditions	Mariana workers presented less favorable living and working conditions and greater vulnerability to common mental disorders; qualitative accounts described social, family, and economic repercussions	2–3	Descriptive and cluster analyses; no conventional multivariable adjustment	Occupational sample; relatively small sample and comparison with another mining community; limited generalizability
	Cavalheiro Paulelli et al., 2022—cross-sectional biomonitoring	Residents of Regência, Povoação, and Campo Grande; n=300	Three affected communities; water, fish/seafood consumption and smoking considered as related exposure factors	Post-disaster biomonitoring	Blood concentrations of 11 elements	Higher blood concentrations of Al, As, Hg, and Ni were observed. Community, drinking water, fish/seafood consumption, and smoking were associated with metal/metalloid levels	1–3	Regression analysis of community, water source, fish/seafood consumption, and smoking in relation to blood metal concentrations	Cross-sectional biomonitoring; no clinical health outcomes; source and timing of exposure cannot be established
	Cavalheiro Paulelli et al., 2023—cross-sectional biomonitoring	Residents of affected communities; n=279	Urinary metals and 8-OHdG	Post-disaster biomonitoring	Urinary As, Cd, Hg, Ni, Pb and 8-OHdG	Urinary As, Cd, and Ni were positively associated with 8-OHdG, whereas Hg and Pb were not	1–3	Bootstrap regression examining associations between urinary metals and 8-OHdG	Cross-sectional design; 8-OHdG is a biomarker rather than a clinical outcome; causal interpretation is limited
Mariana & Brumadinho	Vidal et al., 2024—segmented regression using secondary health system data	Monthly Brazilian public health system data	Pre- and post-disaster periods	24 months before and after each disaster	Hospital admissions, hospitalization expenditure, mortality	A significant change in mortality rate was observed in Brumadinho. Changes in hospitalization indicators were not statistically significant	2–3	Segmented regression of monthly health system data before and after the disasters; no individual level confounder adjustment	Aggregated secondary data; no assessment of individual exposure; possible confounding related to temporal variation

PTSD: Post-Traumatic Stress Disorder. 4.1.2. Mariana.

#### Brumadinho

Following the Córrego do Feijão dam failure in Brumadinho, several studies investigated health conditions among residents using baseline data collected in 2021, approximately two years after the disaster. The Brumadinho Health Project included 3,080 residents aged 12 years or older. In the baseline cohort, hypertension (30.1%), high cholesterol (23.1%), and depression (22.5%) were among the most frequently reported diagnosed conditions (Peixoto et al., 2022). Comparisons with the Metropolitan Region of Belo Horizonte and the state of Minas Gerais were also performed using data from the 2019 National Health Survey (Peixoto et al., 2022).

Mental health was assessed in 2740 adults. Depressive symptoms were the most prevalent psychiatric condition (29.3%), followed by post-traumatic stress symptoms (22.9%) and anxiety symptoms (18.9%). In adjusted analyses, female sex and residence in a mining area were positively associated with several psychiatric outcomes, including symptoms of depression, post-traumatic stress, anxiety, thoughts of death or self-injury, and poor sleep quality (Garcia et al., 2022).

Multimorbidity was investigated among 2777 adults and was identified in 53.8% of participants. After adjustment, multimorbidity was more frequent among women, participants aged 40–59 years and those aged 60 years or older, and residents of areas directly affected by the dam failure. Direct residence in an affected area was associated with multimorbidity (adjusted OR 1.6, 95% CI 1.3–2.0) (Nascimento-Souza et al., 2022).

Respiratory health was assessed in 2,772 adults. The prevalence of asthma was 7.2% and chronic obstructive pulmonary disease was 3.5%, while wheezing, dry cough, and nasal irritation were reported by 8.8%, 23.6%, and 31.8% of participants, respectively. Residents of areas directly affected by the tailings or located in mining areas had greater odds of asthma, while residents of areas directly affected by the tailings reported greater odds of all respiratory symptoms evaluated (Campos et al., 2022).

The use of psychotropic medication was evaluated among 2,805 adults. Overall, 17.2% reported using at least one psychotropic medication during the 15 days preceding the interview. The use of anxiolytics or hypnotics/sedatives was higher among residents of areas directly affected by the tailings, while overall psychotropic drug use was higher among participants who had lost a household member, family member, or friend in the disaster and among those who reported worsening health after the dam failure (Loyola Filho et al., 2022).

Laboratory analyses provided additional evidence of biological alterations. Among the 3,080 participants in the Brumadinho Health Project, laboratory results were available for 2,782 individuals. No significant differences were observed between directly affected and non-exposed participants for most biochemical and hematological parameters. However, participants living in areas with mining activity had higher prevalence of altered HDL cholesterol, high-sensitivity C-reactive protein, and estimated glomerular filtration rate below 60 mL/min/1.73 m^2^ (Maluf et al., 2022).

Human biomonitoring studies provided evidence of internal exposure to potentially toxic elements. Mota et al., (2021) assessed arsenic, cadmium, mercury, lead, and manganese in a representative sample of 3,080 residents aged 12 years or older. Concentrations above reference values were identified in 38.08% of participants for manganese, 33.37% for arsenic, 5.04% for lead, 0.76% for mercury, and 0.17% for cadmium. The authors also identified associations between age and manganese and lead levels, and between place of residence and arsenic levels.

Biomonitoring was also conducted among 217 children aged 0–6 years, of whom 197 provided urine samples. Urinary arsenic and manganese concentrations were higher in some locations not directly affected by the disaster than in directly affected areas. As no clinical health outcome was assessed in this study, these findings represent evidence of internal exposure rather than evidence of a clinical health effect (Santos et al., 2023).

Health system indicators provided an additional population perspective. Macinko et al. (2022) analyzed data from 2,805 adults and compared participants directly affected by the dam failure with individuals who were not directly affected and with residents of an active mining community. Participants in the exposed group had a 15% higher prevalence of a medical consultation in the previous year, whereas no significant differences were observed for hospitalization or recent blood pressure and blood glucose checks. The exposed group also had a higher prevalence of being up to date with recommended vaccinations.

The Brumadinho studies provided evidence across Levels 1–3. Levels 2 and 3 were represented by studies of psychiatric symptoms, multimorbidity, respiratory outcomes, psychotropic drug use, laboratory indicators, and healthcare utilization, while Level 1 was represented by human biomonitoring of potentially toxic elements. Although several studies identified associations between exposure-related variables and health-related outcomes, the predominant use of cross-sectional designs and geographic or indirect exposure proxies limits causal interpretation.

#### Mariana

 Following the Fundão dam failure in 2015, health related evidence was identified from predominantly residential populations, as well as from an occupational defined group of mining workers. In Barra Longa, an exploratory cross-sectional study involving 507 residents from urban and rural areas found that 37% of participants reported worse health conditions compared with the period before the disaster. Among spontaneously reported health problems, respiratory conditions accounted for 40%, skin conditions for 15,8%, and behavior disorders for 11%. Headache, cough, leg pain, pruritus, and anxiety were among the most frequently reported symptoms (Vormittag et al., 2018). The findings represent self-reported health outcomes and do not establish a specific disease related to exposure.

Mental health was also evaluated among mining workers who were working in Mariana when the Fundão dam failed. Comparing 164 miners working in Mariana with miners working in another mining municipality and conducting qualitative interviews with 25 participants, the Mariana workers reported less favorable living and working conditions and were described as more prone to common mental disorders (Motta & Borges, 2021). As this study focused on an occupational population and used another mining community as the comparison group, its findings should be interpreted with specific population context.

Human biomonitoring provided evidence of internal exposure among residents of communities directly affected by the Fundão disaster. The measurement of eleven elements in the blood of 300 participants from Regência, Povoação, and Campo Grande showed elevated exposure levels for aluminum, arsenic, mercury, and nickel. Metal concentrations were associated with community, drinking water, fish and seafood consumption, and smoking habits (Cavalheiro Paulelli et al., 2022).

In a subsequent study involving 279 residents of communities affected by the Fundão dam failure, urinary concentrations of arsenic, cadmium, mercury, nickel, and lead were evaluated together with urinary 8-hydroxy-2'-deoxyguanosine (8-OHdG), a biomarker of oxidative stress and DNA damage. Arsenic, cadmium, and nickel concentrations were positively associated with urinary 8-OHdG, whereas no association was observed for mercury or lead (Cavalheiro Paulelli et al., 2023). This study therefore provides evidence of internal exposure and a biomarker exposure association, but not evidence of a clinical disease outcome or causal effect (Cavalheiro Paulelli et al., 2023).

It was also assessed changes in hospitalization, expenditure, and mortality in Mariana using segmented regression. In Mariana, hospital admissions showed an immediate reduction followed by an upward trend during the 24 months after the disaster, but neither change was statistically significant. Changes in the mortality rate and hospitalization expenditure were also not statistically significant, although temporal patterns were observed (Vidal et al., 2024).

Overall, the evidence from Mariana included Level 1 evidence from human biomonitoring, Level 2 evidence from self-reported health and mental health outcomes, and Level 3 evidence where variables related with exposure were statistically examined in relation to health outcomes or biomarkers. Together, these studies provide indications of health conditions and internal exposure among affected populations, although the heterogeneity of study designs and exposure measures limits the strength of the available evidence. As in Brumadinho, the available studies do not allow firm conclusions regarding causal relationships between the disaster and the reported health outcomes.

#### Synthesis of health-related evidence

Across the two disasters for which health evidence was identified, the available studies covered different stages of the health exposure. Level 1 evidence documented internal exposure through human biomonitoring, including measurements of potentially toxic elements in blood and urine. Level 2 evidence included directly assessed health outcomes and health system indicators, such as psychiatric symptoms, multimorbidity, respiratory conditions, laboratory indicators, psychotropic drug use, hospitalization, mortality, and healthcare utilization. Level 3 evidence was identified when exposure or related exposure variables were statistically examined in relation to health outcomes, biomarkers, or health system indicators. Although several studies reported outcome exposure associations, most were cross-sectional or relied on geographic or indirect exposure indicators, limiting causal interpretation.

Although several studies have identified associations between exposure-related variables and health outcomes or biomarkers, the predominance of cross-sectional designs, geographical indicators of exposure, self-reported outcomes and aggregated data from the health system limits causal interpretation. The available evidence should therefore be interpreted as indicating documented health outcomes, internal exposure and associations between exposure and outcome, rather than causal effects attributable to tailings dam disasters.

### Potential health effects informed by toxicological evidence

The evidence presented in this section represents contextual toxicological evidence and should be distinguished from the direct evidence relating to human health following the disaster, as summarized in Sect. "Evidence on human health following tailing dam disasters". The potential health effects described below are based on established toxicological literature on PTEs and crystalline silica and were not necessarily observed in populations affected by the tailings dam disasters included in this review. This information is provided to contextualize plausible exposure pathways and potential health effects associated with contaminants reported in mining tailings.

In addition, baseline environmental contamination can be a significant confounder when assessing tailings dam disaster impacts on human health. Since tailings dam failures frequently happen in historical, active mining basins, establishing precise background levels is necessary to isolate disaster damage from legacy contamination (Buch et al., 2023). The Rio Doce basin is a clear example: following the Fundão collapse, investigations in the upper basin revealed that non-affected soils and river sediments already carried elevated baseline levels of arsenic, barium, cobalt, chromium, copper, nickel, and zinc, frequently exceeding regional quality reference values prior to the accident (Davila et al., 2020). However, this elevated geochemical background cannot be interpreted as evidence that the mine tailings are not associated with PTEs across the entire basin. The study of Davila et al. (2020) was restricted to the upper Rio Doce basin, where the pre-existing signature of the historical *Quadrilátero Ferrífero* mining district is strongest. In this sense, a local background cannot be extrapolated to the entire system affected by a disaster of the magnitude of Fundão, which released more than 50 million m^3^ of tailings and travelled over 660 km to the Atlantic coast (Gomes et al., 2017; Queiroz et al., 2018). Indeed, Gomes et al. (2017) showed an increase in the concentrations of PTEs after the disaster compared to the baseline contents in the estuarine region, far from any legacy mining source. Taken together, these findings indicate that baselines are meaningful only at the local scale and cannot represent the full footprint of a tailings dam failure, which must be assessed along the entire environmental gradient.

A further limitation concerns the scarcity of pre-disaster data on the human population. Baseline biomonitoring (e.g., blood, urine, or hair PTE levels) is rarely available for communities in mining basins before a failure occurs (Ondayo et al., 2024). In addition, baseline health conditions and structural socioeconomic disadvantages characteristic of mining territories substantially worsen post-disaster physical and mental morbidity (Li et al., 2023; Peixoto et al., 2026; Vidal et al., 2025). Populations in long-established mining communities endure historical exposures and resource deficits that may amplify post-event harm and impede long-term recovery (Cools et al., 2022; Kim et al., 2025). While environmental monitoring of tailings or wastewater is occasionally mandatory, regulatory frameworks frequently lack statutory requirements to evaluate internal human burdens prior to catastrophic failures (Salazar et al., 2024).These factors reinforce the need for integrated, longitudinal surveillance that establishes both environmental and human baselines, enabling disaster-related effects to be distinguished from pre-existing contamination and morbidity when interpreting chronic exposure.

Among the PTEs most frequently reported in the tailings, As deserves particular attention because of its widespread occurrence across different mineral commodities and its broad spectrum of toxicological effects. Chronic As exposure has been associated with peripheral neuropathy, hyperpigmentation, hyperkeratosis, hypertension, atherosclerosis, gastrointestinal disorders, and hepatic and respiratory damage (Balali-Mood et al., 2021; Jomova et al., 2025; Luo et al., 2012; Shen et al., 2013).

Cadmium exposure has been associated with renal dysfunction, bone demineralization, osteoporosis, and chronic obstructive pulmonary disease (COPD), whereas Pb primarily affects the nervous, hematological, and renal systems (Balali-Mood et al., 2021; Dongre et al., 2011; Fay et al., 2018; Wang et al., 2013). Mercury mainly affects neurological and renal function and may also induce gastrointestinal disorders (Bottino et al., 2016; Chen et al., 2019; Zhang et al., 2020).

Although Fe, Mn and Al are naturally occurring elements and essential in specific physiological processes, excessive exposure may produce important toxicological effects. Iron overload has been associated with liver disease, cardiomyopathy and diabetes, whereas Mn exposure has been linked to manganism, neurodevelopmental impairment and neurological dysfunction. Al exposure has been associated with oxidative stress, renal dysfunction and neurodegenerative disorders (Coetzee et al., 2016; Exley, 2013; Jomova et al., 2025; Lucchini et al., 2022; McLaren et al., 2008). Finally, inhalation of crystalline silica (SiO_2_), commonly present in mining dust, represents an additional occupational and environmental concern because of its association with silicosis, COPD, chronic kidney disease, autoimmune disorders and lung cancer (Howlett et al., 2023; Leung et al., 2012; NIOSH, 2011; Souza et al., 2021;).

### Health implications and public health surveillance priorities

Evidence from Mariana and Brumadinho indicates that the consequences of tailings dam failures extend well beyond the acute phase of the disaster. Higher prevalence or occurrence of cardiovascular and respiratory conditions, anxiety, depression, and other mental health outcomes has been reported in some populations affected by tailings dam disasters (Campos et al., 2022; Garcia et al., 2022; Nascimento-Souza et al., 2022; Peixoto et al., 2022). These findings may reflect the combined influence of multiple factors, including exposure to PTEs, socioeconomic disruption, environmental degradation, loss of livelihoods, displacement, and psychological trauma (Leppold et al., 2022; Motta & Borges, 2021; Noal et al., 2019). However, the available studies do not allow the relative contribution of these factors to be determined or causal relationship to be established.

Communicable diseases have also been reported following these disasters. In Mariana and Brumadinho, increased dengue incidence was associated with environmental disturbances, disruption of sanitation systems and changes in local ecological conditions that may favor mosquito breeding (Freitas et al., 2022; Kossoff et al., 2014). These observations illustrate the multifactorial nature of health impacts following disasters, in which environmental, social and factors related to exposure may interact. However, the available evidence does not allow these relationships to be interpreted as causal effects arising exclusively from dam failures.

The evidence summarised in Table 2 was heterogeneous, covering outcomes relating to mental health, respiratory conditions, multimorbidity, laboratory indicators, human biomonitoring, the use of psychotropic medicines and health system indicators. This heterogeneity, together with the frequent use of cross-sectional designs and indirect measures of exposure, limits direct comparison between studies and causal interpretation.

These findings support the need for integrated public health surveillance following tailings dam disasters, combining environmental monitoring, periodic biomonitoring, epidemiological follow-up, and assessment of relevant physical and mental health outcomes. Such surveillance may help characterize changes in exposure and health over time and improve identification of populations requiring continued monitoring and support.

### Strengths, limitations and knowledge gaps

**Fig. 5:**
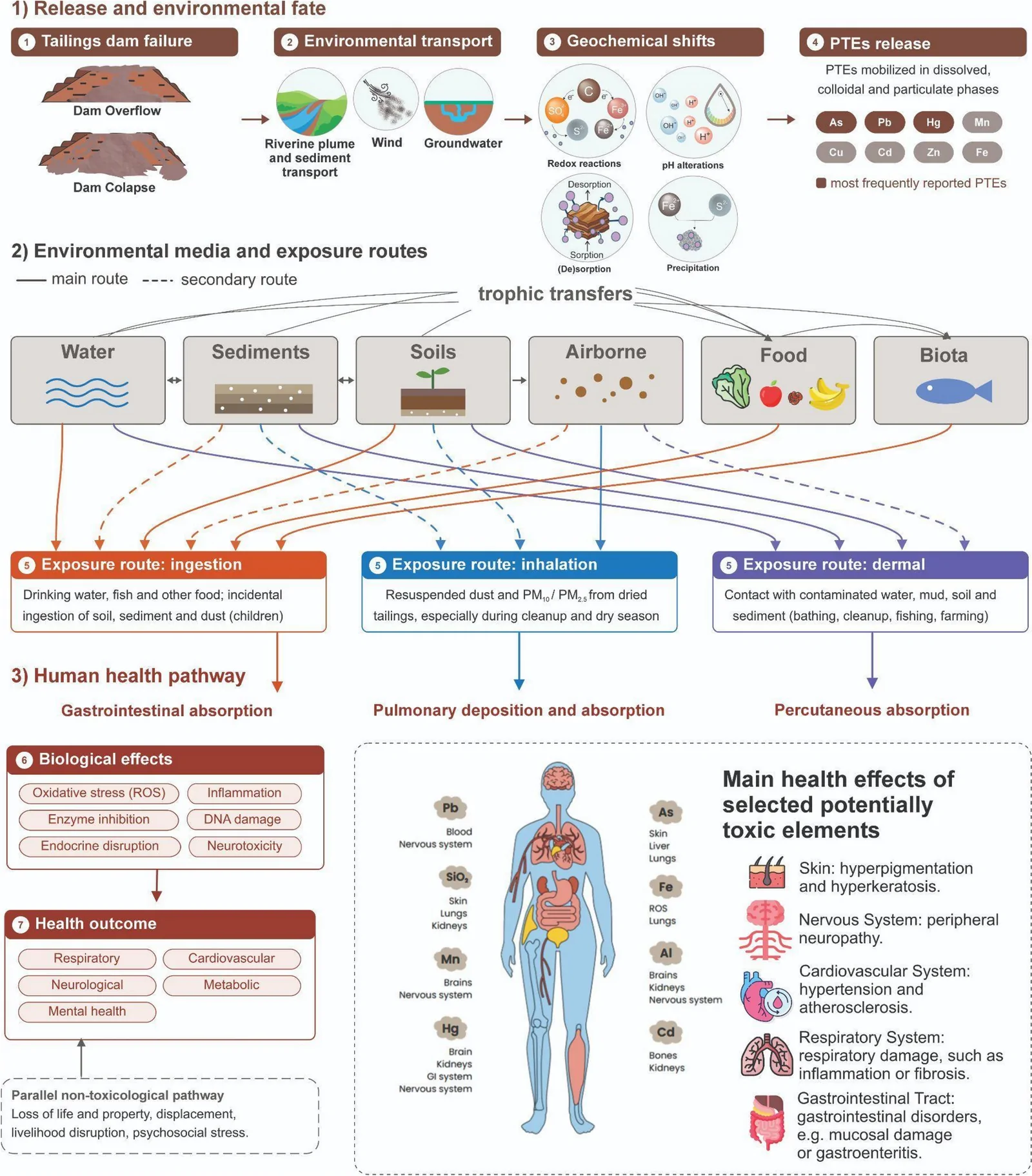
Conceptual framework linking tailings dam failure to human health outcomes. The diagram integrates three domains. (1) Release and environmental fate: a tailings dam failure (overflow or collapse) drives environmental transport (riverine plume and sediment transport, wind, groundwater), geochemical shifts (redox reactions, pH alterations, (de)sorption, precipitation) and the release of potentially toxic elements (PTEs) in dissolved, colloidal and particulate phases; As, Pb, Hg and Mn (brown) were the most frequently reported PTEs. (2) Environmental media and exposure routes: PTEs partition among six media (water, sediments, soils, airborne particulate matter, food and biota) connected by inter-compartmental exchange and trophic transfers, reaching humans through three principal exposure routes — ingestion, inhalation and dermal contact (solid lines = main route; dashed lines = secondary route). (3) Human health pathway: each route leads to internal dose via gastrointestinal, pulmonary or percutaneous absorption, followed by biological effects and health outcomes; a parallel non-toxicological pathway (loss of life and property, displacement, livelihood disruption, psychosocial stress) also contributes to mental-health outcomes. The body panel summarizes the main organs and systems affected by selected elements — lead (Pb), quartz (SiO_2_), manganese (Mn), mercury (Hg), arsenic (As), iron (Fe), aluminium (Al) and cadmium (Cd). PTEs: potentially toxic elements; PM_10_/PM_2.5_: particulate matter ≤ 10 µm and ≤ 2.5 µm; ROS: reactive oxygen species (oxidative stress); GI: gastrointestinal

To our knowledge, this review represents the first attempt to integrate, on a global scale, data on mining tailings dam disasters with the corresponding evidence on human health outcomes in the past decade. We applied a scoping approach combining structured database searches (Web of Science, Scopus, MEDLINE) with an iterative, event-specific search strategy and grey literature sources (WISE Uranium Project, UNEP, GRID-Arendal, WHO), allowing the identification of disasters and health evidence that would likely have been missed by generic database searches alone. The classification of evidence into three levels (biomonitoring, health outcomes/health system indicators, and exposure-outcome associations) provided a structured framework for characterizing the type and the available evidence. A conceptual framework (Fig. 5) linked PTEs release, environmental fate, exposure routes and health outcomes integrating environmental and toxicological evidence.

At the same time, several limitations inherent to the scope and data sources of this review should be acknowledged. First, reliance on the WISE Uranium Project chronology, institutional reports (UNEP, GRID-Arendal, WHO) and targeted grey-literature searches means the disaster inventory could be biased toward larger, more catastrophic, or more widely publicized events; smaller or less visible failures, particularly in low-resource or less-monitored jurisdictions, may be underrepresented, and as noted for the event inventory, records for several disasters were incomplete or inconsistent, so aggregate figures for tailings volumes, deaths, missing persons and displacement should be interpreted as minimum or approximate estimates rather than precise counts.

The chemical characterization of released tailings was available for only 10 of the 49 disasters identified, and the absence of reported PTE data for the remaining events cannot be interpreted as evidence that those tailings did not contain potentially toxic elements, limiting the completeness of the exposure picture across the full inventory. Most critically for the health-related objective of this review, only two of the 49 disasters (Mariana and Brumadinho) had published population-based health studies; this extreme concentration of evidence reflects a knowledge gap.

Finally, pre-existing geochemical and health baselines in historical mining territories, combined with the predominance of cross-sectional designs, self-reported outcomes, and geographic or indirect exposure indicators in the available studies, introduces ecological confounding that limits the ability to isolate disaster-attributable effects from pre-existing environmental and socioeconomic conditions.

## Conclusions

This review identified 49 tailings dam disasters reported worldwide between 2015 and 2025, resulting in 670 reported deaths, 112 injuries, with additional reports of missing and displaced people. The concentration of incidents in regions such as Minas Gerais (Brazil), Kachin State (Myanmar), Potosí (Bolivia), mining regions in China, and major gold- and copper-producing areas in Peru and Mexico highlights geographical clusters of reported tailings dam disasters in regions with intensive extractive activities and multiple tailings storage facilities. Gold, copper, jade, and iron were among the most frequently reported mineral commodities most frequently involved in these disasters, while arsenic, lead, mercury, manganese, and zinc were the most commonly reported potentially toxic elements.

The evidence compiled in this review demonstrates that tailings dam failures represent not only environmental disasters but also important public health challenges. Exposure to PTEs and other substances released or mobilized during some of these events has been investigated in relation to adverse health outcomes, including respiratory, cardiovascular, neurological, and mental health disorders. However, one of the most important findings of this review is the substantial lack of epidemiological evidence: although 49 disasters were identified, only two events (Mariana and Brumadinho) have been investigated in population-based studies evaluating human health following the events. This highlights a major global knowledge gap concerning the consequences of mining disasters for exposed populations.

Future efforts should prioritize long-term environmental and health surveillance programs, including biomonitoring of potentially toxic elements, epidemiological studies, and standardized assessment protocols. At the same time, the adoption of safer tailings management practices, improved monitoring systems, and more robust regulatory frameworks will be essential to reducing the occurrence of future disasters and their impacts on human health and the environment.

## Supplementary Information

Below is the link to the electronic supplementary material.

Supplementary file1 (DOC 73 KB)

## Data Availability

No datasets were generated or analysed during the current study.
